# On the Role of the Carboxyl Group to the Protective Effect of *o*-dihydroxybenzoic Acids to *Saccharomyces cerevisiae* Cells upon Induced Oxidative Stress

**DOI:** 10.3390/antiox11010161

**Published:** 2022-01-14

**Authors:** Nikolaos Nenadis, Efi Samara, Fani Th. Mantzouridou

**Affiliations:** Laboratory of Food Chemistry and Technology, School of Chemistry, Aristotle University of Thessaloniki, 54124 Thessaloniki, Greece; niknen@chem.auth.gr (N.N.); efisamara2010@hotmail.com (E.S.)

**Keywords:** protocatechuic acid, pyrocatechuic acid, dihydroxybenzoic acids, *Saccharomyces cerevisiae*, oxidative stress, DFT, radical scavenging, hydrogen peroxide, cumene hydroperoxide

## Abstract

In the present work, the role of the carboxyl group of *o*-dihydroxybenzoic acids (pyrocatechuic, 2,3-diOH-BA and protocatechuic, 3,4-diOH-BA) on the protection against induced oxidative stress in *Saccharomyces cerevisiae* was examined. Catechol (3,4-diOH-B) was included for comparison. Cell survival, antioxidant enzyme activities, and TBARS level were used to evaluate the efficiency upon the stress induced by H_2_O_2_ or cumene hydroperoxide. Theoretical calculation of atomic charge values, dipole moment, and a set of indices relevant to the redox properties of the compounds was also carried out in the liquid phase (water). Irrespective of the oxidant used, 2,3-diOH-BA required by far the lowest concentration (3–5 μM) to facilitate cell survival. The two acids did not activate catalase but reduced superoxide dismutase activity (3,4-diOH-BA>2,3-diOH-BA). TBARS assay showed an antioxidant effect only when H_2_O_2_ was used; equal activity for the two acids and inferior to that of 3,4-diOH B. Overall, theoretical and experimental findings suggest that the 2,3-diOH-BA high activity should be governed by metal chelation. In the case of 3,4-diOH BA, radical scavenging increases, and chelation capacity decreases. The lack of carboxyl moiety (3,4-diOH B) improves to radical scavenging, interaction with lipophilic free radicals, and antioxidant enzymes. The present study adds to our knowledge of the antioxidant mechanism of dietary phenols in biological systems.

## 1. Introduction

Free radicals are produced in the body during metabolism and contribute to signal transduction, the expression of genes, and the activation of various receptors. Nevertheless, exposure to environmental factors (i.e., UV, ionizing radiations, pollutants, and heavy metals), adoption of various medications (i.e., antiblastic drugs) and habits (e.g., smoking) combined with an unbalanced diet can facilitate free radical overproduction [1]. The latter cannot be tackled by the endogenous antioxidant system, thus leading to oxidative stress. Such a condition has been associated with a high risk for the formation of chronic diseases (non-communicable) that account for the 71% of total deaths globally [2]. Epidemiological, clinical, and nutritional studies have highlighted that a diet rich in foods of plant origin with high content of phenolic compounds may be beneficial toward lowering the risk of formation of such diseases [3] due to their antioxidant activities, taking into account their role in the plant defense from various types of stress [4].

Among phenolic compounds, about 30% of free or bound forms are phenolic acids [5]. The simpler members are hydroxybenzoic ones, which can be found in various medicinal herbs, tea, rosaceous fruits, wines, potatoes, avocados, and Aspergillus-fermented soy products [6], but can also be formed from flavonoids via the activity of gut microbiota [7]. Some bearing a catechol group, namely 2,3- and 3,4- dihydroxy benzoic acids (2,3-diOH-BA, 3,4-diOH-BA), have been identified in plasma and urine after consumption of plant-derived foods [6] and possess a broad spectrum of biological activities in vivo, including antioxidant [8,9]. From the mechanistic point of view, these acids seem to be active radical scavengers in vitro due to the presence of the catechol moiety, a prerequisite for high antioxidant activity according to structure–radical scavenging activity (SAR) principles [10]. Despite the various advantages of such tests in SAR studies, as highlighted in the past by Haenen and co-workers [11], and their wide applicability to study the antioxidant potential of food and extracts, they are not relevant to biological systems. Therefore, issues and challenges are raised regarding the understanding of the role of phenol structural characteristics in oxidative stress amelioration [5]. Although these issues have been highlighted for almost 15 years, in vivo mechanistic information is still scarce, as recently reported by Martins et al. [12]. To assist in closing such a gap, phytochemical evaluation of antioxidant activity is proposed in cancer cells (PC-12 and HepG2) or yeast such as *Saccharomyces cerevisiae*. The latter presents a series of advantages as a model organism to study biological oxidative stress in humans granted that “yeast and human gene are highly conserved” [13]. 

Given the above, and taking into account that comparative studies on phenolic compounds (natural or synthetic) employing this model have been carried out mainly for molecules differing significantly in structure so far [14,15,16,17,18,19,20], it was quite challenging to examine the antioxidant performance of the 2,3-diOH-BA and 3,4-diOH-BA in the particular model for the first time. To highlight the contribution of the carboxyl moiety, the more lipophilic catechol (3,4-diOH-B) was co-examined. Before this, the two acids and 3,4-diOH-B were characterized with regards to their physicochemical characteristics that may influence their antioxidant potential using quantum chemical calculations [21,22]. The computations were carried out in a model simulating solvation in water, that is, the environment where the phenols are expected to be mainly located in the cells due to their polarity. With the present study, we aim to add to the existing knowledge of the mechanism of action of dietary phenols in biological systems.

## 2. Materials and Methods

### 2.1. Chemicals

Hydrogen peroxide (H_2_O_2_) (35% *w*/*w*), potassium dihydrogen phosphate (KH_2_PO_4_) (≥99.5%), and dipotassium phosphate (K_2_HPO_4_) (≥99%) were purchased from Riedel-de Haën (Seelze, Germany). 3,4-diOH-B (≥98% purity) was purchased from Fluka Chemie GmbH (Buchs, Switzerland). 2,3-diOH-BA (≥99.5% purity), 3,4-diOH-BA (≥99.5% purity), cumene hydroperoxide (CumOOH) (≥80% purity), ethylenediaminetetraacetic acid (EDTA) calcium disodium salt (≥97%), phenylmethanesulfonyl fluoride (PMSF, solution ~0.1 M in ethanol), Bradford reagent comprised of the Coomassie Brilliant Blue G-250 (CBBG) dye in phosphoric acid and methanol, (-)-riboflavin (≥98%), L-methionine (≥98%), 2-thiobarbituric acid (ΤΒΑ) (≥98%), bovine serum albumin (BSA) (≥98%) and 1,1,3,3-tetra ethoxy propane (TEP) (≥96%) were obtained from Sigma Chemical Co. (St. Louis, MO, USA). Culture media contained D-glucose monohydrate (Panreac Quimica S.A., Barcelona, Spain), yeast extract (Merck, Darmstadt, Germany), and soy peptone (LabM Limited, Bury, UK). Absolute ethanol (99% purity), trichloroacetic acid (TCA) (≥99.5%), and ultrahigh-purity water were from Chem-Lab (Zedelgem, Belgium). All other common reagents and solvents were of the appropriate purity from various suppliers. 

### 2.2. Biological Material

*S. cerevisiae* strain used in this study was the wild-type strain BY4741(ATCC 4040002) (MATa; his3Δ1; leu2Δ0; met15Δ0; ura3Δ0), generously provided by Dr. Antonios Makris (Inst. of Applied Biosciences (INEB) of the National Center for Research and Technological Development (CERTH), Thessaloniki, Greece), maintained at 4 °C on YPDA (yeast extract 10 g/L, peptone 20 g/L, glucose 20 g/L, agar 20 g/L) slants. Catalase (CAT) from bovine liver (lyophilized powder, 2.000–5.000 units/mg protein) and lyticase from *Arthrobacter luteus* (lyophilized powder, ≥200 units/mg solid) were purchased from Sigma Chemical Co. (St. Louis, MO, USA).

### 2.3. S. cerevisiae Inoculum Preparation

Yeast cells stored on YPDA slants were activated in the same medium by maintaining consecutive transfers. The inoculum was prepared by transferring a loopful of yeast cells from the agar slants to 500 mL hydrophobic cotton-stopped Erlenmeyer flasks containing 100 mL of YPD liquid medium (pH 5.5) with the same composition as for YPDA except for agar. Flasks were incubated in a shaking incubator at 120 rpm and 28 °C for 16 h to a final OD_600_ value of approximately 1.0 (corresponding to the mid-exponential growth phase of yeast cells). 

### 2.4. Assay for Screening Induction of S. cerevisiae Oxidative Stress Resistance

The yeast cell survival assay was performed according to Erkekoglou et al. [23] with slight modifications. Specifically, yeast cells at the mid-exponential phase (prepared as described in § 2.3) were transferred in fresh YPD medium (at an OD_600_ value of 0.2) containing two different oxidative agents, H_2_O_2_ (5 mM) or CumOOH (150 μM), in the absence/presence of the phenolic compounds, namely 2,3-diOH-BA, 3,4-diOH-BA and 3,4-diOH-B (2–200 µM), and incubated for 2 h at 28 °C on a rotary shaker at 120 rpm (treated cells). All of the above phenolic solutions were prepared in ethanol and sterilized by passing through a 0.45 μm membrane filter (Schleicher Schnell, Dassel, Germany) before inoculation. Τhe control cells (untreated) and the oxidant-treated cells without supplementation of the test compounds were supplemented with an equivalent amount of ethanol for comparison reasons. 

Cell viability, expressed as log_10_ CFU/mL, was measured by serially diluting 1-mL aliquots of cell suspensions and plating on a YPDA medium. The plates were incubated at 28 °C for 72 h, and the final colony count was taken as the average of at least 3 plates for the dilution containing 30–300 colonies per plate. The number of colonies observed in the control plate (untreated cells) was set as 100% *S. cerevisiae* and resistance was expressed as the survival percentage with regards to the control calculated as:

$$\mathrm{Survival}\;\left(\%\right)=\frac{{\left(\frac{\mathrm{CFU}}{\mathrm{mL}}\mathrm{YPD}\right)}_{\mathrm{treated}\;\mathrm{cells}}}{{\left(\frac{\mathrm{CFU}}{\mathrm{mL}}\;\mathrm{YPD}\right)}_{\mathrm{untreated}\;\mathrm{cells}}}\times 100$$

All assays were carried out at least in triplicate.

### 2.5. Preparation of Cell Extracts

Cells were harvested by centrifugation at 10,000× *g* for 20 min (4 °C) and washed twice in 50 mM potassium phosphate buffer (KPi, pH 7.0). After washing, the wet biomass (0.2–0.3 g) was suspended in 4 mL lysis buffer (1 mM PMSF, 1 mM EDTA, 50 mM KPi, pH 7.0) containing 1mg/mL lyticase (20 units/mL), and then incubated at 37 °C for 45 min under gentle agitation. The suspension was sonicated using an ultrasonic processor UP50H (50 watts, 30 kHz) with a MS7 standard probe (tip-diameter of 7 mm), in an ice bath, for 2–5 min at 50% duty cycles of sonication (active interval) (s) (0.5 s on/0.5 s off). Cell debris was removed by centrifugation for 20 min at 10.000× *g* and the supernatant (crude cell extract) was kept on ice for immediate use. 

### 2.6. Total Protein Content Determination

Total protein content (TPC) in the crude cell extract was determined by the Bradford protein assay [24] and the absorbance of CBBG was measured at 595 nm using the Hitachi UV–VIS (U-2000) spectrophotometer (Tokyo, Japan). BSA was used as a standard in a concentration range of 0.07–1.4 mg/mL of protein. According to the standard calibration curve, the value of TPC for each crude cell extract was presented as mg/mL. Data are presented as mean (±SD) of three independent measurements (CV% = 1.3, *n* = 5).

### 2.7. Determination of Antioxidant Enzyme Activities

CAT activity was determined spectrophotometrically at 240 nm following degradation of H_2_O_2_ in a medium containing 50 mM KPi (pH 7.0), 0.5 mM EDTA, 10 mM H_2_O_2,_ and 40–100 µL of crude cell extract [25]. One unit of CAT activity is defined as the amount of the enzyme that catalyzed the degradation of 1 mmol of H_2_O_2_ per min and results are presented as the mean ± SD CAT activity (units/mg protein) from at least four independent measurements (CV% = 5.3, *n* = 5). 

Superoxide dismutase (SOD) activity was measured based on its ability to inhibit the photochemical reduction of NBT [26]. The reaction mixture consisted of potassium phosphate buffer (50 mmol/L, pH 7.8), methionine (10 mmol/L), EDTA (0.1 µmol/L), NBT (1 mmol/L), and riboflavin (2.4 µmol/L) and the required amount of crude cell extract (50–100 µL). Sample test tubes and control test tubes (without samples) were placed in an aluminum foil-lined box under 15 W fluorescent light for 10 min. The reading of solution absorbance was carried out at 560 nm using the Hitachi UV–VIS (U-2000) spectrophotometer. A non-irradiated complete reaction mixture served as a blank. SOD activity is the measure of NBT reduction in light without protein minus NBT reduction with protein. One unit of SOD is defined as the amount of enzyme required to cause 50% inhibition of the NBT photoreduction rate and results are presented as the mean ± SD SOD activity (units/mg protein) from at least four independent measurements (CV% = 3.8, *n* = 5).

### 2.8. TBARS Assay

The TBARS (thiobarbituric acid-reactive substances) assay was performed as described by Steels et al. [27] with modifications. Next, 10% (*w*/*v*) trichloroacetic acid (final concentration) was added to the crude cell extracts, which were then centrifuged for 20 min at 10,000× *g*. The supernatant was mixed with 0.1 mL EDTA (0.1 M) and 0.6 mL 1% (*w*/*v*) thiobarbituric acid in 0.05 M NaOH. Blank samples contained lysis buffer instead of supernatant. The reaction mixture was incubated in a water bath (90 °C) for 15 min and, after rapid cooling, the absorption was measured at 532 nm using the Hitachi UV–VIS (U-2000) spectrophotometer. The results are expressed as nmoles of malondialdehyde (MDA) equivalents/mg protein and then assigned as the mean ± SD of the ratio between the treated cells and control cells (untreated) from three independent measurements (CV% = 1.1, *n* = 5). If the TBARS concentration is lower in oxidant-treated cells supplemented with phenolic compounds than that in treated cells without added phenolic compounds, the phenolic compound has acted as an antioxidant. 

### 2.9. Statistical Analysis

The results were statistically evaluated by one-way analysis of variance (ANOVA). Comparison of experimental mean values was performed with Duncan’s Multiple Range test (*p* ≤ 0.05 confidence level). The software SPSS 20 (SPSS Inc., Chicago, IL, USA) was used to statistically process the results.

### 2.10. Computational Details

All calculations for tested compounds were performed by the Gaussian 09W rev. A.02- SMP program [28]. The B3LYP functional was used for geometry optimization and computation of harmonic vibrational frequencies using the 6-31+G(d,p) basis set (unrestricted B3LYP for the resulting radicals). Single-point electronic energies were then obtained using the 6-311++G(2d,2p) basis set. Implicit solvent effects (water) were taken into account with the aid of integral equation formalism of the polarized continuum model (IEF-PCM) and the united atom for Hartree−Fock (UAHF) solvation radii [29,30]. Employing the electronic energies (298 K) at 6-311++G(2d,2p) and thermal contributions to enthalpy obtained at 6-31+G(d,p), the corresponding thermodynamic parameters that characterize the different radical mechanisms by phenols, as exemplified in Figure S1 for 3,4-diOH-BA were calculated as follows: The bond dissociation enthalpy (BDE) values that characterize hydrogen atom transfer (HAT) activity according to the equation:

BDE= H_r_ + H_h_ − H_p_ (1)

where Hr is the enthalpy of the radical generated by H atom abstraction, H_h_ is the enthalpy of the H atom, and H_p_ is the enthalpy of the parent molecule. 

The ionization potential (IP) values that characterize single-electron transfer−proton transfer (SET-PT) efficiency, according to the equation:

IP = H_cr_ + H_e_ − H_p_ (2)

where H_p_ and H_cr_ stand for the enthalpy of the parent molecule and the corresponding cation radical generated after electron transfer, whereas He− is the enthalpy of the electron. 

The O−H PDE (proton dissociation enthalpy) values by using the formula:

PDE= H_r_ + H_pr_ − H_cr_ (3)

in which H_r_ is the enthalpy for radical generated after proton dissociation, H_pr_ is the enthalpy for proton, and H_cr_ is the enthalpy for the cation radical. 

Proton affinity (PA) values that define the first step of the SPLET (sequential proton loss electron transfer) mechanism according to the formula:

PA = H_a_ + H_pr_ − H_p_ (4)

where H_a_ is the enthalpy of the anion generated after deprotonation, H_pr_ is the enthalpy of the proton, and H_p_ is the enthalpy of the parent molecule. Then, electron transfer enthalpy (ETE) values for the anions via the equation:

ETE= H_r_ + H_e_- − H_a_ (5)

where H_r_, H_a_, and H_e_- refer to the enthalpy of the phenoxy radical, the anion, and the electron, respectively. 

No spin contamination was found for radicals, with ⟨S2⟩ values of about 0.750 in all cases. For the calculation of the various aforementioned molecular descriptors, the enthalpy values of hydrogen atom (water, −0.49992348 hartree) were computed, whereas the experimental values were adopted for hydrogen cation (water, −0.41516 hartree) and electron (water, −0.0492478 hartree) [31,32]. All the computed values of molecular descriptors were expressed in kcal/mol (1 hartree = 627.509 kcal/mol). 

HOMO, LUMO energies, atomic charge values, and dipole moment values were obtained in the liquid phase (water). Other descriptors, namely the chemical hardness, η = (I − A)/2, the softness of the molecule S = 1/(2η), the electronegativity of the molecule χ = (I + A)/2, the electrophilicity of the molecule ω = μ^2^/(2η), and the maximum number of electrons that an electrophile may obtain ΔN_max_ = −µ/η were calculated using I = −E_HOMO_, A = −E_LUMO_ and the electrochemical potential μ = −χ [22]. 

Calculation of the Log*P* values, which express the partitioning of the phenols in an *n*-octanol/water system, was based on the CS ChemDraw Ultra and chemical structure drawing standard 1985–2003 program (CambridgeSoft Corporation, Cambridge, MA, USA). 

## 3. Results

### 3.1. Theoretical Study

Because the pKa value of the carboxyl group is 2.93 and 4.42 for 2,3-diOH-BA and 3,4-diOH-BA, respectively [33], and under the experimental conditions the pH value (extra-/intra- cellular) is expected to be higher, the respective moiety was considered ionized in the computations.

Structure optimization at B3LYP/6-31+G(d,p) level of theory showed that the most stable conformers were those bearing intramolecular bonds. In the case of 2,3-diOH-BA, due to the configuration of substituents, two intramolecular bonds were formed, so that no free phenolic hydrogen atom was available as shown in the Supplementary Material (Figure S2). 

The introduction of the -COO^−^ was predicted to significantly increase hydrophilicity based on Log*P* values (2,3-diOH-BA: 0.44, 3,4-diOH-BA: 0.45, 3,4-diOH-B: 1.09). A similar trend was obtained in terms of another index of molecular polarity, namely the computed dipole moment values in the liquid phase (2,3-diOH-BA: 11.14 D, 3,4-diOH-BA: 16.69 D, 3,4-diOH-B: 3.75 D). Comparing the two acids, 3,4-diOH-BA was predicted as more polar than 2,3-diOH-BA, especially in terms of dipole moment values. 

Except for the hydrophilicity of the compounds, the introduction of the ionized carboxyl group was expected to affect the charge and its distribution in the atoms of the molecules as shown in the Supplementary Material (Figure S3). The latter may influence the interaction of the compounds with transition metals such as Fe^2+,^ which decompose hydroperoxides in biological systems through redox reactions. As observed, 3,4-diOH-BA and 3,4-diOH-B may have one site for chelating transition metals (HO-C_3_-C_4_−OH), with the negative charge distribution being lower in the latter compound. In the 2,3-diOH-BA the corresponding sites were two, with the negative charge mostly located in the -OOC-C_1_-C_2_−OH part of the molecule (salicyl group), followed by that in the HO-C_2_-C_3_−OH part (catechol group). This is in line with the comments of Hirpaye and Rao [34] on the complexation sites of the particular compound with various divalent metal cations (Co^2+^, Ni^2+^, Cu^2+^, Zn^2+^). 

The reactivity of the tested compounds was first examined via the calculation of a set of indices derived from the values of their frontier orbitals (Table 1).

Based on the corresponding indices, it was evident that the introduction of the -COO^−^ is expected to improve electron donation and more when participating in an intramolecular bond (2,3-diOH-BA).

According to the set of thermodynamic descriptors that characterize the scavenging of free radicals (Table 2), it is evident that the presence of the -COO^−^ should reduce the hydrogen atom transfer efficiency (BDE values) or the ability to form phenoxy ions (PA values). 

The lowest efficiency is predicted for 2,3-diOH BA, since the two −OH groups in the aromatic ring participate in intramolecular hydrogen bonds (highest BDE and PA values). An opposite trend is observed in terms of electron donation (IP, ETE values) which is in agreement with the evidence provided in Table 1. However, from a thermodynamic point of view, SPLET should be the preferable mechanism of action, as the PA values that determine its occurrence are the lowest among all calculated descriptors. This is in accord with the proposal made by Perez-Gonazlez et al. [35] for dihydroxybenzoic acids at physiological pH, who used a kinetic approach instead of a thermodynamic one. Thus, the radical scavenging order of activity predicted should be 3,4-diOH-B> 3,4-diOH-BA>2,3-diOH-BA.

### 3.2. Experimental Study

#### 3.2.1. Cytoprotective Effect of 2,3-diOH-BA, 3,4-diOH-BA, and 3,4-diOH-B in H_2_O_2_-Stressed S. cerevisiae Cells

**Figure 1:**
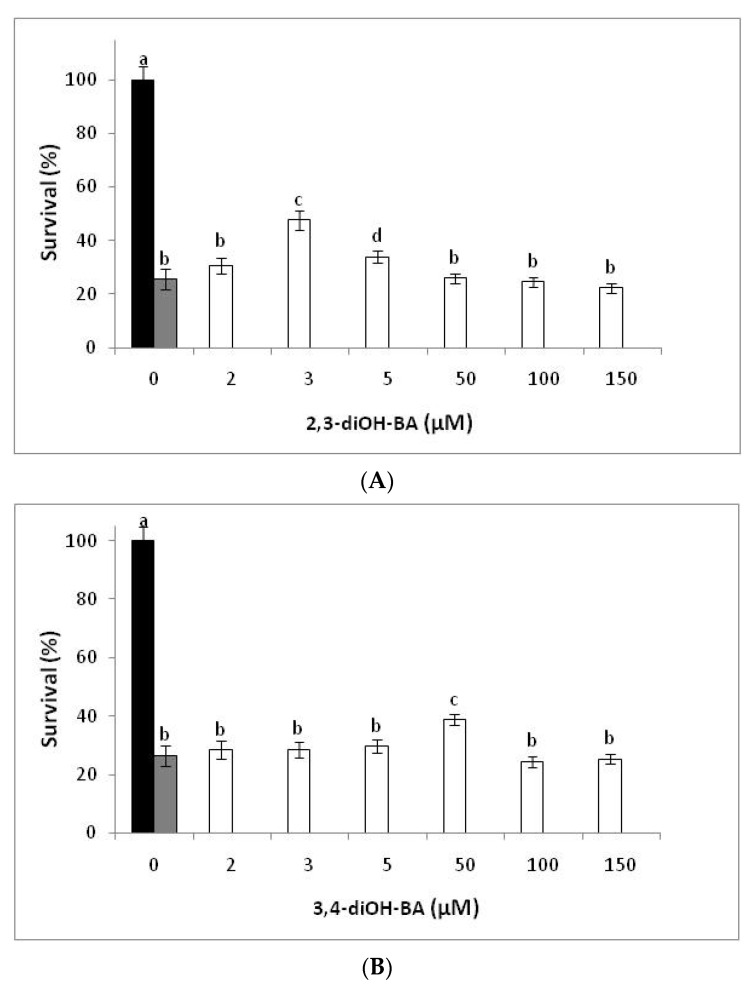

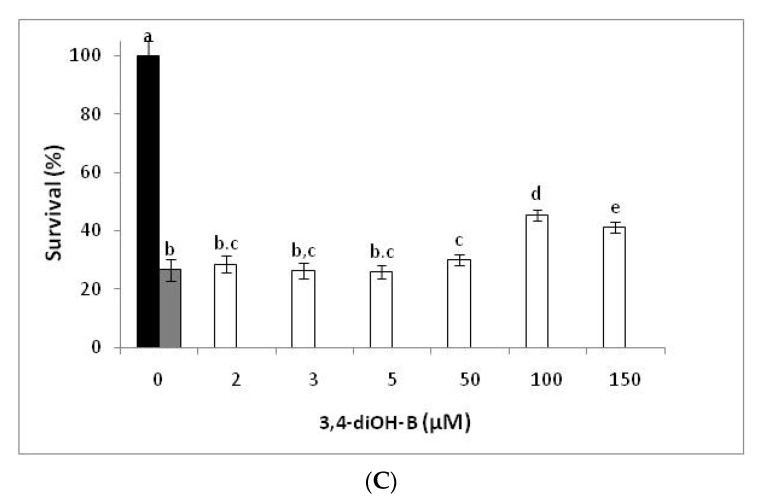
Effect of the tested phenolic compounds on survival rates of *S. cerevisiae* BY4741 cells treated with H_2_O_2_ (5 mM). Black bar means untreated cells, gray bar means cells treated only with H_2_O_2_, and white bars mean cells treated with H_2_O_2_ + 2,3-diOH-BA (**A**), 3,4-diOH-BA (**B**), and 3,4-diOH-B (**C**). Data represent the mean values ± SD of at least three independent experiments. Different lowercase letters above the bars indicate a statistically significant difference at *p* < 0.05.

The cytoprotective effect of the tested phenols was examined in *S. cerevisiae* BY4741 cells upon exposure to 5 mM H_2_O_2_ for 2 h. According to findings, the addition of the phenolic compounds in a wide range of concentrations (2–200 µM) was not toxic for the wild-type strain BY4741 and the cells continued to reach 100% tolerance (data not shown). The values of survival rates for six concentrations (2, 3, 5, 50, 100, and 150 µM) and cells treated only with H_2_O_2_ are representatively shown in Figure 1. 

Yeast cells showed sensitivity to the oxidizing agent and only 26% of the yeast population was able to survive the induced oxidative stress. Upon addition of the compounds, an increase in the recovery of the surviving cells above the oxidant-induced death indicated positive antioxidant activity [23]. In the presence of 2,3-diOH-BA at 3 µM, 48% of the yeast population survived, whereas no protection was observed for the oxidant-induced cells at concentrations of this phenolic compound higher than 5 µM. 3,4-diOH-BA exerted significant protection (39% of yeast population) at a ~17-fold higher concentration (50 µM). The levels of 3 and 50 µM for 2,3-diOH-BA and 3,4-diOH-BA, respectively, correspond to the lowest ones that induced a significant improvement in cell survival. This was evidenced upon examination of their addition in the various concentrations. 3,4-diOH-B was found to function equally well to the *ο*-dihydroxybenzoic acid anions but at 100 µM (46% of yeast population). At lower levels, namely 50 µM, the improvement of cell survival (30% of yeast population) was minor, whereas at too-low ones (3 and 5 µM, respectively) non-significant changes (26% of the yeast population, respectively) was found. Thus, the introduction of -COO^−^ in catechol, participating in an intramolecular hydrogen bond as in the 2,3-diOH BA, was critical for the improved survival of the cells.

#### 3.2.2. Cytoprotective Effect of 2,3-diOH-BA, 3,4-diOH-BA, and 3,4-diOH-B in CumOOH-Stressed *S. cerevisiae* Cells

**Figure 2:**
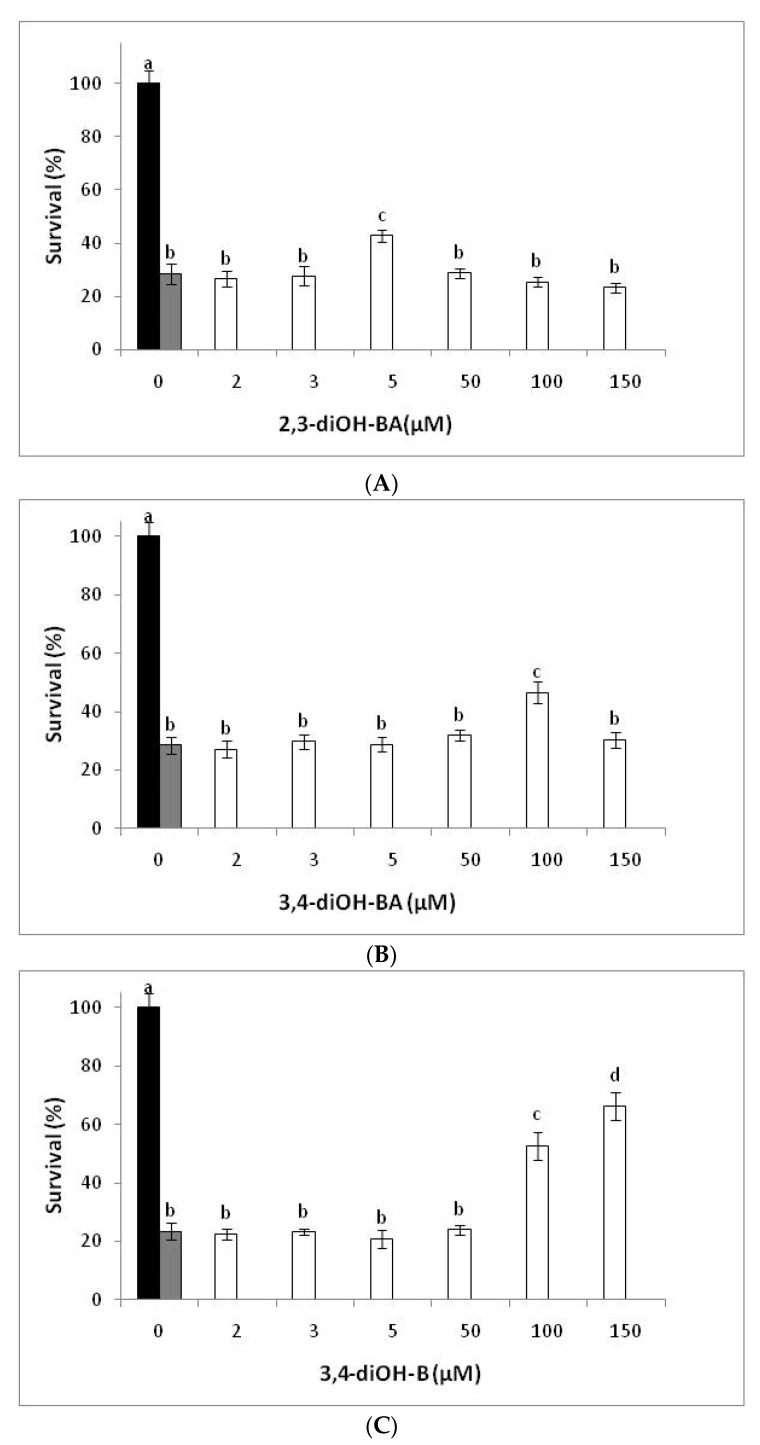
Effect of the tested phenolic compounds on survival rates of *S. cerevisiae* BY4741 cells treated with CumOOH (150 µM). Black bar means untreated cells, gray bar means cells treated only with CumOOH and white bars mean cells treated with CumOOH +2,3-diOH-BA (**A**), 3,4-diOH-BA (**B**), and 3,4-diOH-B (**C**). Data represent the mean values ± SD of at least three independent experiments. Different lowercase letters above the bars indicate a statistically significant difference at *p* < 0.05.

*S. cerevisiae* cells possess distinct response mechanisms to maintain protection against different reactive oxygen species (ROS) [36]. In this view, the effectiveness of a compound against one oxidant may differ compared to another. Thus, in the present study, the tested compounds were examined regarding their protection against the oxidative stress induced by a larger-sized and lipophilic oxidizing agent, the CumOOH (Figure 2) at a typical concentration (150 µM) for the examination of the oxidative stress responses in yeast cells employing particular oxidizing agents [20].

Similar to H_2_O_2_-treated cells, 2,3-diOH-BA addition upon CumOOH treatment was highly effective at low concentration. Specifically, a 42% recovery of surviving cells was found at 5 μM. At higher concentrations of 2,3-diOH-BA, no protection was observed (Figure 2A). 3,4-diOH-BA and 3,4-diOH-B did not exert any protection at concentrations <50 µM. Τhe minimum effective concentrations of 3,4-diOH-BA and that of 3,4-diOH-B was 100 µM providing survival of 47 and 53%, respectively.

#### 3.2.3. Effect of 2,3-diOH-BA, 3,4-diOH-BA, and 3,4-diOH-B on the Activity of Antioxidant Enzymes

The activity of CAT and SOD was determined to examine whether the protective effect of 2,3-diOH-BA, 3,4-diOH-BA, and 3,4-diOH-B was associated with these antioxidant enzymes. In the absence/presence of the tested phenols at the lowest effective concentration, the CAT and SOD activity of control cells extract and those treated with H_2_O_2_ or CumOOH are shown in Table 3.

When compared to control cells, the activity of CAT in cells exposed to oxidants increased significantly. Specifically, H_2_O_2_ (5 mM) stimulated the enzyme activity by ~3-fold, while CumOOH (150 µM) stimulated it by ~4-fold (22.7 and 27.1 vs. 7 units/mg protein). The two oxidants also stimulated the SOD activity, both by ~2-fold compared to the control cells (32.3 and 34.8 vs. 17 units/mg protein). However, regardless of the oxidant, the level of stimulation of CAT and SOD activity was not enough to protect the exponentially growing yeast cells against the ROS under the current experimental conditions (26–28% cell survival) (Figure 1 and Figure 2).

As shown in Table 3, no significant changes in CAT activity were observed upon the addition of the tested phenolic compounds to H_2_O_2_-treated cells. Thus, the protective effect of phenolics addition in the presence of H_2_O_2_ was not related to CAT activity. In CumOOH-treated cells, a significant increase in CAT enzyme activity (34.1 vs. 27.1 units/mg protein) was induced only upon 3,4-diOH-B supplementation. When the activity of the SOD enzyme was evaluated, it was observed that the addition of the phenolic compounds reduced its activity (Table 3). This reduction was highly potentiated in the case of 3,4-diOH-B. Therefore, it could be argued that the tested phenolic compounds could act as “SOD-like” enzymes, and thus as radical scavengers [37,38]. Moreover, that the introduction of a -COO^−^ group in the aromatic ring favored less SOD activity reduction, especially when participating in an intramolecular hydrogen bond as in the case of 2,3-diOH BA.

#### 3.2.4. Effect of 2,3-diOH-BA, 3,4-diOH-BA, and 3,4-diOH-B on TBARS Level

Next, TBARS ratio values were measured. Table 4 shows that in the H_2_O_2_-treated cells without adding phenolic compounds, the ratio value was two. This value is in agreement with that reported in published studies, where *S. cerevisiae* cells were subjected to H_2_O_2_ stress under the same experimental conditions e.g., [27].

Exposure of the cells to H_2_O_2_ in the presence of the phenolic acids led to a significant decrease in the mean value of the TBARS ratio (1.8) and an even greater decrease in the presence of 3,4-diOH-B (1.6) (Table 4). The phenolic compounds did not restore the original state of the cells, despite lowering oxidative stress, implying that H_2_O_2_ was still harmful to cells. Upon CumOOH treatment in the present study, the TBARS concentration was the same in both treated and control cells (TBARS ratio equal to one). Thus, it was not possible to comment on the antioxidant activity of the tested compounds.

## 4. Discussion

In the present work, the effect of exposure of *S. cerevisiae* cells to 2,3-diOH-BA and 3,4-diOH-BA in the presence of a hydrophilic (H_2_O_2_) or a lipophilic (CumOOH) oxidizing agent was investigated concerning specific oxidative markers. To comment further on the role of the carboxyl group (ionized form under experimental conditions) to the protective effect to the *S. cerevisiae* cells of these two acids, a comparison was made to 3,4-diOH-B. The latter is more hydrophobic, and thus may better diffuse through the cell membrane. The cell death observed at elevated levels of peroxides can be attributed to the formation of highly toxic free radicals into the cell through the catalytic reaction of the redox-active transition metal ions, such as the iron available in free form or linked with intracellular molecules at low-affinity sites (labile iron pool) [39]. Such catalytic action applies not only to the H_2_O_2_ but also to the synthetic ones used in biological studies such as CumOOH [40]. 2,3-diOH-BA was found as the most effective cytoprotective compound, regardless of the type of oxidizing agent used. Among the other two phenolic compounds examined, at 50 μM, 3,4-diOH-BA was more effective against the cytotoxic activity of H_2_O_2_ than 3,4-diOH-B (39 vs. 30%), whereas the latter was slightly more effective against CumOOH at 100 μM (53 vs. 47%).

The main mechanisms that are involved in the in vivo protective action of natural phenolic compounds against oxidative stress are (a) prevention of reactive free radicals formation through transition metal ion chelation, (b) scavenging of free radicals, and (c) induction of the expression of antioxidant enzymes [41] with the prerequisite to cross biological membranes and reach biological targets within cells [42].

Regarding the prevention of the formation of reactive free radicals through transition metal ion chelation, all three compounds may act like this as the presence of a catechol group contributes positively to their binding [43]. The 2,3-diOH BA should present a higher chelation efficiency due to the concomitant presence of the salicyl group in the structure of the acid except for the catechol group, as supported by the computed charge values in the atoms (Figure S3). According to the latter values, the particular structural feature should be more important than the catechol due to the higher negative charge values suggesting greater interaction with the metal ions. Because 2,3-diOH BA anion is very polar—as is the Fe^2+^, which may decompose the hydroperoxides (both types)—its efficiency which was higher by far compared to that of the other two phenolic compounds under both H_2_O_2_ and CumOOH stress suggest that metal chelation should be decisive for its performance. This is further supported by the fact that (i) 2,3-diOH-BA has previously been identified as a very efficient iron ion chelator, for which reason it has been exploited for removing excess iron from the body [44]; (ii) 3,4-diOH-BA is a rather weak Fe^2+^ chelator, as evidenced by its experimental binding constants compared to those of other phenols or phenolic acids at pH 7.4 [45]. Accepting that the computed atomic charge values are useful to comment on metal ion chelation of the test compounds, it could also be argued from Figure S3 that 3,4-diOH BA may probably bind slightly better the metal ions than 3,4-diOH B due to the higher negative charge values in the oxygen atoms of the catechol group of the former. Consequently, chelation may influence the estimated activity of 3,4-diOH BA in the cell system more than that of 3,4-diOH B.

Concerning the prevention through radical scavenging, the computational indices suggested that this pathway should contribute less to the experimental findings observed in the case of 2,3-diOH BA. A higher contribution should be to 3,4-di OH BA activity and even more to that of 3,4-diOH B. The inferior antioxidant potency expected for 2,3-diOH BA through radical scavenging adds further to the fact that it should mainly act through metal chelation as reported by other authors on antioxidant activity studies involving transition metals [43]. Similarly, considering that 3,4-diOH BA is an inferior radical scavenger to 3,4-diOH B, its twofold lower minimum effective concentration required for cell survival in H_2_O_2_-treated cells compared to catechol could be related to its slightly better Fe^2+^ chelation efficiency. The tendency of 3,4-diOH B to act preferably as a radical scavenger rather than a chelator is depicted by the lower TBARS values recorded upon H_2_O_2_-induced stress, although these values may be related to oxidation products from a radical attack to different biomolecules except for lipids [46]. The better performance of 3,4-diOH BA upon H_2_O_2_ vs. CumOOH may be related to the fact that the polar oxidant forms hydroxyl radicals due to decomposition by Fe^2+^, which can be deactivated by molecules in its vicinity as its diffusion is slower than the radicals’ half-life [47]. Therefore, 3,4-diOH BA, which is more polar than 3,4-diOH B, may be located closer to the site of formation of the hydroxyl radical. On the other hand, in the case of peroxyl radicals that are lipophilic and do not only react with vicinal compounds, the chelation of Fe^2+^ to prevent decomposition of CumOOH may account mostly for the protective effect found for 3,4-diOH BA. The 3,4-diOH B being less polar and acting preferably as a radical scavenger may explain its higher concentration required to become efficient against H_2_O_2_ and its better performance upon oxidation with CumOOH.

The cytoprotective action of *o*-dihydroxy-phenolic compounds against oxidative stress has been also associated with the induction of intracellular antioxidant enzymes [5,13]. In the present work, no activation of CAT by the tested phenolic compounds was observed in cells cultured under conditions of increased oxidative stress in the presence of H_2_O_2_. Considering the improvement observed in survival from the oxidative stress of yeast cells supplemented with the phenolic compounds (39–48% vs. 26%) it can be deduced that the treatment of cells with a high dose of H_2_O_2_ could have caused enzyme (CAT) inactivation by the substrate [48]. Therefore, the role of the carboxyl group was not evident. In contrast, in CumOOH-treated cells, a significant increase in CAT enzyme activity (34.1 vs. 27.1 units/mg protein) was induced upon 3,4-diOH-B supplementation. This was in accord with reports highlighting that the organic peroxides do not inactivate yeast antioxidant enzymes [48]. Thus, the elevated survival of CumOOH-treated cells supplemented with 3,4-diOH-B (53% vs. 28%) against oxidative stress (Figure 2) could be expected to be partially caused by the stimulation of CAT activity. This finding could be related to its higher diffusion through cell membrane, thus permitting interaction with the lipophilic free radicals at the site of oxidation. Then, having converted to the more electrophilic quinone (ΔN_max_ = 3.36) may enhance the CAT activity by activating the transcription factor Yap1e [49,50]. Concerning SOD activity, a reduction was observed in the presence of the phenolic compounds. The reduction was lower in phenolic acids with the lowest observed for 2,3-diOH-BA, where the carboxyl group participates in an intramolecular hydrogen bond. All three compounds presented a SOD-like activity, a property also reported for other phenolic compounds in literature [38]. The order of decreasing the SOD activity is in line with their radical scavenging efficiency predicted in Section 3.1 in terms of PA values (Table 2). The poorer SOD activity of the two acids, and mainly of the 2,3-diOH BA, add further to the fact that the corresponding acid should mainly act as a metal chelator.

## 5. Conclusions

The findings of the present work showed that the presence of a carboxyl group and its location in a benzene ring with a catechol moiety (*o*-dihydroxybenzoic acids) can significantly affect the antioxidant activity at a molecular level in a biological system. When its introduction leads to the formation of a salicylic group as in 2,3-diOH-BA, a high efficiency is induced. This was shown by the too-low levels of addition of the acid required for a positive effect compared to 3,4-diOH-BA and 3,4-diOH-B, regardless of the of the oxidant (H_2_O_2_, CumOOH) used. The experimental and theoretical findings, including literature information, support that 2,3-diOH BA antioxidant potency should be governed by metal chelation, whereas radical scavenging and antioxidant enzyme activation should poorly contribute. In the case of 3,4-diOH BA acid, poorer metal chelating capacity is expected; thus, the mechanism of action should be characterized mostly by radical scavenging. The absence of the carboxyl group further adds to the radical scavenging character (3,4-diOH B), and improves interaction with lipophilic radicals and antioxidant enzymes. Despite the complexity of the interactions in the cellular system that make it difficult to precisely interpret the mechanism of action involved, the present study adds to the existing knowledge of the structure–antioxidant activity relationship of dietary phenols in biological systems.

## Figures and Tables

**Table 1:** Computed reactivity descriptors of tested phenols derived from frontier orbitals in the liquid phase (water) at B3LYP/6-31+G(d,p)//B3LYP/6-311++G(2d,2p) level.

	χ	H	s	ω	ΔN_max_	HOΜO	LUMO	ΔE_LUMO−HOMO_
2,3-diOH-BA *	134.5	55.9	3521.9	55.2	1.41	−134.5	−22.7	111.8
3,4-diOH-BA *	135.9	58.7	3351.1	50.6	1.31	−135.9	−18.3	117.5
3,4-diOH-B	137.1	64.7	3040.8	40.0	1.12	−137.1	−7.6	129.5

* carboxyl group was considered ionized.

**Table 2:** Computed thermodynamic descriptors of tested phenols characterizing the radical scavenging activity in the liquid phase (water) at B3LYP/6-31+G(d,p)//B3LYP/6-311++G(2d,2p) level.

	BDE	IP	PDE	PA	ETE
2,3-diOH-BA *	82.8 **/76.7 ***	93.2	11.90	35.2	69.9
3,4-diOH-BA *	80.6 **/76.0 ***	95.3	7.62	31.4	71.5
3,4-diOH-B	76.6 **/74.9 ***	96.4	2.52	27.3	71.6

* carboxyl group was considered ionized; ** enthalpy of C4−OH; *** enthalpy to form a quinone upon sequential hydrogen atom transfer.

**Table 3:** Catalase (CAT) and superoxide dismutase (SOD) activity in *S. cerevisiae* BY4741 cells treated with H_2_O_2_ (5 mM) or CumOOH (150 μM) in the absence/presence of the tested phenolic compounds.

Oxidizing Agent	Phenolic Compound (μM)	CAT ^1^	SOD ^1^
-	-	7.0 ^A^ ± 0.5	17.0 ^A^ ± 1.5
H_2_O_2_	-	22.7 ^aB^ ± 1.2	32.3 ^aB^ ± 2.6
	2,3-diOH-BA (3)	23.6 ^a^ ± 5.2	25.8 ^b^ ± 1.2
	3,4-diOH-BA (50)	26.6 ^a^ ± 4.9	12.6 ^c^ ± 1.5
	3,4-diOH-B (100)	22.1 ^a^ ± 4.3	2.9 ^d^ ± 0.8
CumOOH	-	27.1 ^a,b,C^ ± 3.4	34.8 ^aB^ ± 4.5
	2,3-diOH-BA (5)	31.3 ^b,c^ ± 4.0	24.7 ^b^ ± 3.8
	3,4-diOH-BA (100)	24.5 ^a^ ± 4.0	6.0 ^c^ ± 1.7
	3,4-diOH-B (100)	34.1 ^c^ ± 4.5	3.2 ^c^ ± 0.5

^1^ CAT and SOD activities are expressed in units/mg total protein of the cell extract. Data represent the mean values ± SD of at least three independent experiments. Different lowercase letters indicate significant differences between treatments with the same oxidizing agent in the absence/presence of the phenolic compounds; different uppercase letters indicate statistically significant differences between untreated cells and cells treated only with H_2_O_2_ or CumOOH (*p* < 0.05).

**Table 4:** TBARS levels (expressed as µmol malondialdehyde per mg protein) in *S. cerevisiae* BY4741 cells treated with H_2_O_2_ (5 mM) or CumOOH (150 μM) in the absence/presence of the tested phenolic compounds.

Oxidizing Agent	Phenolic Compound (μM)	Ratio ^1^
H_2_O_2_	-	2.0 ^aA^ ± 0.04
	2,3-diOH-BA (3)	1.8 ^c^ ± 0.02
	3,4-diOH-BA (50)	1.8 ^c^ ± 0.01
	3,4-diOH-B (100)	1.6 ^b^ ± 0.02
CumOOH	-	1.1 ^aB^ ± 0.03
	2,3-diOH-BA (5)	1.1 ^a^ ± 0.03
	3,4-diOH-BA (100)	1.0 ^c^ ± 0.01
	3,4-diOH-B (100)	1.0 ^b^ ± 0.03

^1^ The results were expressed as a ratio between TBARS levels (expressed as nmoles of malondialdehyde equivalents/mg protein) in cells exposed to the oxidizing agents in the absence/presence of phenolic compounds relative to that in the untreated (control) cells. Data represent the mean values ± SD of at least three independent experiments. Different lowercase letters indicate statistically significant difference at *p* < 0.05 between treatments with the same oxidizing agent in the absence/presence of the phenolic compounds; different uppercase letters indicate statistically significant differences between treatments with H_2_O_2_ and CumOOH in the absence of the phenolic compounds (*p* < 0.05).

## Data Availability

All data are contained within the article.

## Supplementary Materials

The following are available online at https://www.mdpi.com/article/10.3390/antiox11010161/s1: Figure S1: Thermodynamic parameters characterizing the 3,4-diOH-BA scavenging of free radicals according to hydrogen atom transfer (HAT), single-electron transfer−proton transfer (SET-PT) and sequential proton loss electron transfer (SPLET); Figure S2: Optimized structures of the most stable conformers of tested compounds at B3LYP/6-31+G(d,p) level of theory (aqueous phase); Figure S3: Computed atomic charge values in the tested compounds at B3LYP/6-31+G(d,p)//B3LYP/6-311++(2d,2p) level of theory (aqueous phase).
