# Supplementary material for: On the Role of the Carboxyl Group to the Protective Effect of o-dihydroxybenzoic Acids to Saccharomyces cerevisiae Cells upon Induced Oxidative Stress
Source: Antioxidants (Basel). 2022 Jan 14;11(1):161. doi: 10.3390/antiox11010161 (PMC8773101; doi:10.3390/antiox11010161)
Supplement: Supplementary file 1 [file antioxidants-11-00161-s001.zip › antioxidants-1519805-supplementary.pdf]

On the Role of the Carboxyl Group to the Protective Effect of *o*-dihydroxybenzoic Acids to *Saccharomyces cerevisiae* Cells upon Induced Oxidative Stress

Nikolaos Nenadis, Efi Samara and Fani Th. Mantzouridou*

Laboratory of Food Chemistry and Technology, School of Chemistry, Aristotle University of Thessaloniki, 54124, Thessaloniki, Greece; niknen@chem.auth.gr (N.N.); efisamara2010@hotmail.com (E.S.)
* Correspondence: fmantz@chem.auth.gr (F.Th.M); Tel.: (+30-2310-997774)

Supplementary material

Figure S1: Thermodynamic parameters characterizing the 3,4-diOH-BA scavenging of free radicals according to hydrogen atom transfer (HAT), single-electron transfer–proton transfer (SET-PT) and sequential proton loss electron transfer (SPLET)

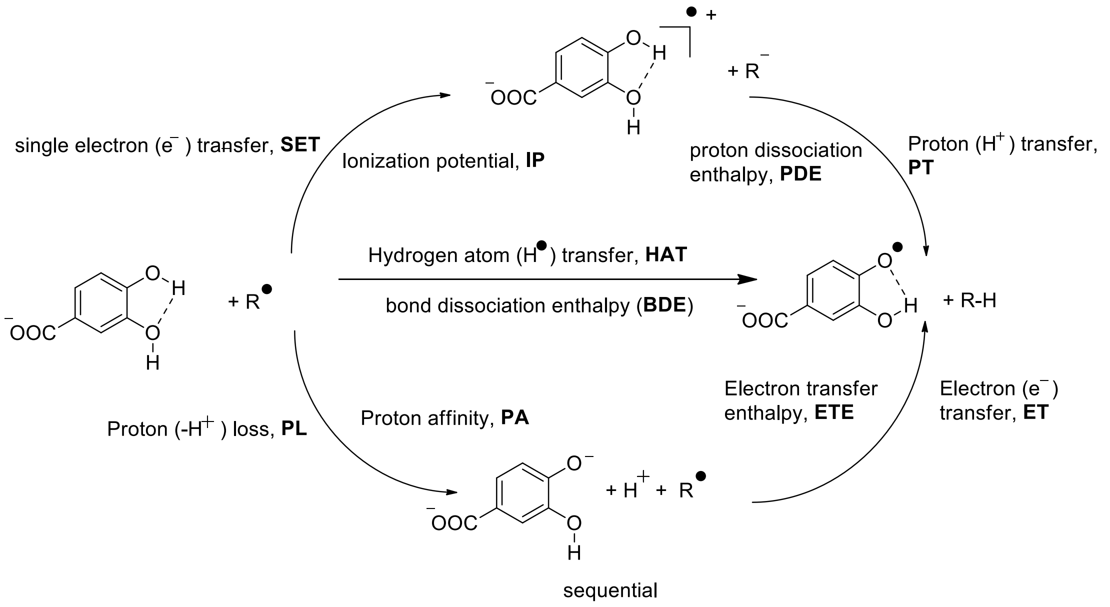

For formula calculation of the thermodynamic parameters see sector 2.10 of the manuscript

Figure S2: Optimized structures of the most stable conformers of tested compounds at B3LYP/6-31+G(d,p) level of theory (aqueous phase)

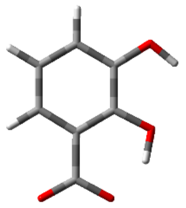

2,3-diOH-BA

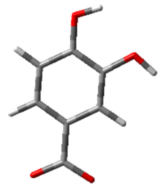

3,4-diOH-BA

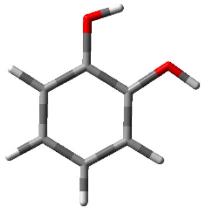

3,4-diOH-B

Figure S3: Computed atomic charge values in the tested compounds at B3LYP/6-31+G(d,p)//B3LYP/6-311++(2d,2p) level of theory (aqueous phase)

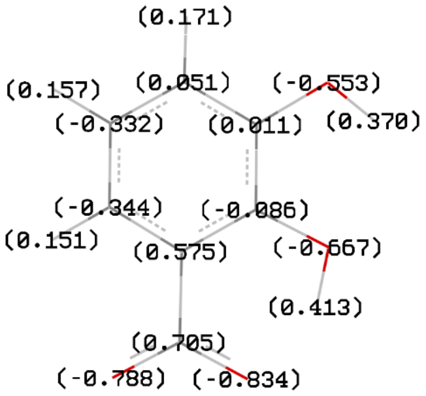

2,3-diOH-BA

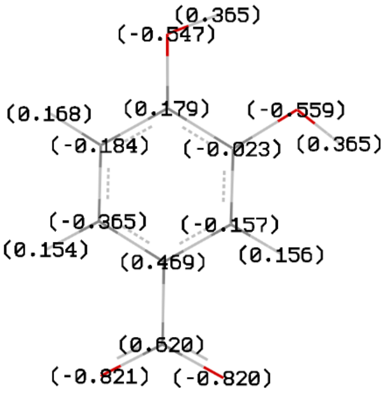

3,4-diOH-BA

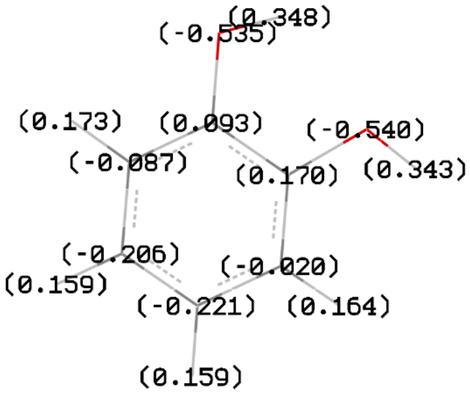

3,4-diOH-B
